# Healthcare workers’ freedom of movement in times of pandemics: an emerging norm of customary international law

**DOI:** 10.1186/s12992-023-00985-y

**Published:** 2023-11-10

**Authors:** Andrés Constantin, Aliya Sternstein

**Affiliations:** 1 O’Neill Institute for National and Global Health Law, Georgetown University Law Center, Washington, DC USA; 2 Georgetown University Law Center, Washington, DC USA

**Keywords:** Healthcare workers, Customary international law, Pandemic treaty, Health workers mobility

## Abstract

**Background:**

A shortage of healthcare workers can hinder the ability to prepare for and respond to global security threats caused by diseases that are prone to pandemics. During the COVID-19 pandemic, the shortage of healthcare workers became a growing concern worldwide. Recognizing these challenges, countries adopted measures to ensure healthcare workers’ freedom of movement in the face of the COVID-19 pandemic. As the WHO continues the negotiation process to reform the 2005 International Health Regulations and to adopt a new Pandemic Treaty, with one key provision relating to healthcare workers’ mobility, questions remain as to whether States will actually adopt a binding international legal instrument or whether its effectiveness will be watered down by the intrinsic vulnerabilities of an international legal system that has (too) often been unable to tame geopolitical interests. Considering these challenges, we assessed the emergence of a norm of customary international law allowing the free movement of healthcare workers during pandemics.

**Methods:**

Our study examined the laws and policies adopted during the COVID-19 pandemic concerning healthcare workers’ mobility in 10 countries, representing all continents. The country selection was based on regional representation and a preliminary analysis indicating their early adoption of measures related to healthcare workers’ mobility. Temporal limits were set. To gather relevant data, we employed various methods including research databases, media sources, and the COVID-19 Law Lab database.

**Results:**

Our research identified and assessed instances of state practice and evidence of *opinio juris* to determine whether a norm of customary international law mandating states to ensure healthcare workers’ freedom of movement during pandemics exists.

The findings indicate a strong consensus towards ensuring the free movement of healthcare workers in times of pandemics as a way to respond to outbreaks of disease. Within months, Argentina, Colombia, Kenya, South Africa, India, Japan, Spain, the United Kingdom, Canada, and the United States, ten nations representing most regions of the world, recognized, as law, the practice of excluding healthcare workers from prohibitions on movement.

**Conclusion:**

Ultimately, this discussion is critical for global health because if a norm does exist in this regard, it will further strengthen pandemic legal preparedness efforts. As such, it becomes clear that the reform of the 2005 International Health Regulations and/or the adoption of a new pandemic treaty will bolster the strength of this emerging norm of customary international law and crystallize it. These legal instruments would propel a norm that is already in the process of formulation into existence. Thus, crystallizing a norm that is otherwise emerging among states.

## Background

Over the past three years, the world witnessed the devastating impact of a global health crisis, which has led to shortages of medical supplies, equipment, and hospital beds, among other critical resources [1]. The COVID-19 pandemic exposed the weaknesses of our healthcare systems and the crucial role that healthcare workers play in them [2].

Despite increased globalization, regulations governing professions are not standardized across all professions and geographical areas [3]. However, the General Agreement on Trade in Services (GATS) has played a role in decreasing obstacles to labor movement between countries and in reducing disparities in education standards [4]. The GATS also regards healthcare workers as tradeable goods, similar to other commodities, and encourages countries to compete in trading health services [5]. Accordingly, medical labor markets can have a significant influence on the provision of healthcare services and how they affect patient outcomes [6].

Back in 2016, the Global Strategy on Human Resources for Health (GSHRH): Workforce 2030 predicted that there will be a shortage of 18 million healthcare professionals by 2030, particularly in low- and lower-middle-income nations [7, 8]. The Dublin Declaration on Human Resources for Health suggested that approximately 40 million additional healthcare workers will be needed by 2030, but the world may fall short by 18 million, which is more than one in five of the 80 million required [9].

For instance, in Africa, out of the 47 World Health Organization (WHO) member states, only 4 countries—Seychelles, Namibia, Mauritius, and South Africa—had densities of physicians, nurses, and midwives per 1,000 people that exceeded 4.45, while the remaining 43 countries had a regional density of 1.55 [10]. The benchmark for the ideal number of health workers (a combination of 13 professions) per 10,000 population is 134.23 [11]. The WHO has identified 55 countries, including 37 in Africa, as vulnerable to having an insufficient number of health workers to achieve target 3.8 of the United Nations Sustainable Development Goals on achieving universal health coverage (UHC) by 2030 [12].

Regardless of economic development level, countries face challenges in educating, employing, deploying, retaining, and ensuring the productivity of their workforce [13]. For example, the United States could experience a shortage of up to 124,000 physicians, including 48,000 primary care physicians, by 2034, according to the Association of American Medical Colleges [14]. To fulfill the increasing demand for healthcare services, the United States needs over 3.2 million more healthcare workers, including nursing assistants, medical assistants, and home health aides, in the next five years [15].

A shortage of healthcare workers can hinder the ability to prepare for and respond to global security threats caused by diseases that are prone to epidemics, such as avian flu, Severe Acute Respiratory Syndrome (SARS), and hemorrhagic fevers, as well as natural and human-made disasters [16]. During the COVID-19 pandemic, the shortage of healthcare workers became a growing concern worldwide, especially as infections from the Omicron variant surged [17, 18].

Shortages of healthcare workers had an adverse effect on patient care in both developed and developing nations [17, 19], with the uneven distribution of healthcare workers raising concerns about the fairness and accessibility of healthcare services around the world [5].

Recognizing these challenges and after learning from previous experiences [16], countries worldwide adopted measures to ensure healthcare workers’ freedom of movement in the face of the COVID-19 pandemic.

Healthcare workers’ freedom of movement ultimately was key in the response to the COVID-19 pandemic as it allowed for the sharing of knowledge and expertise, as well as the ability to provide healthcare in areas where it was needed most. As the pandemic spread rapidly across the globe, healthcare systems were overwhelmed and in need of additional resources, including healthcare workers. The ability of healthcare workers to move freely across borders allowed for their deployment to hotspots and under-resourced communities. Healthcare workers’ mobility allowed for the sharing of information and best practices across borders. Over the course of the pandemic, countries and regions developed different strategies for managing the outbreak, such as testing protocols, contact tracing, and treatment methods. The ability of healthcare workers to move freely across borders allowed them to share this information and best practices, which helped to improve the overall response to the pandemic.

As the WHO continues the negotiation process to reform the 2005 International Health Regulations and to adopt a new Pandemic Treaty, with one key provision relating to healthcare workers’ mobility, questions remain as to whether States will actually adopt a binding international legal instrument or whether its effectiveness will be watered down by the intrinsic vulnerabilities of an international legal system that has (too) often been unable to tame geopolitical interests [20].

Considering these challenges, in this paper, we assess the emergence of a norm of customary international law allowing the free movement of healthcare workers during pandemics. Our research identified and assessed instances of state practice and evidence of *opinio juris* to determine whether a norm of customary international law mandating states to ensure healthcare workers’ freedom of movement during pandemics exists. Ultimately, this discussion is critical for global health because if a norm does exist in this regard, it will further strengthen pandemic legal preparedness efforts.

In Part II, we outline the methods of our study. In Part III we present the findings of our study. In Part IV, we discuss these findings suggesting that regardless of whether the world forges a pandemic treaty, there is a strong consensus and state practice aiming towards ensuring healthcare workers’ freedom of movement during pandemics as a norm of customary international law that is binding on all nations. In Part V, we conclude.

## Methods

The aim of our study was to examine the laws and policies adopted during the COVID-19 pandemic concerning healthcare workers' mobility. To achieve this, we focused on 10 countries, representing all continents, namely Argentina, Colombia, Kenya, South Africa, India, Japan, Spain, the United Kingdom, Canada, and the United States of America. The selection of these countries was based on regional representation and a preliminary analysis indicating their early adoption of measures related to healthcare workers’ mobility.

We set temporal limits for our assessment, specifically focusing on March and April 2020, immediately following the World Health Organization's declaration of a global pandemic. To gather relevant data, we employed various methods including research databases, media sources, and the COVID-19 Law Lab database.

We thoroughly reviewed and assessed government-published or government-broadcast diplomatic statements, international conduct and commitments, correspondence, verbal statements, and guidelines. In cases where official government content was unavailable, we considered multiple press accounts that indicated the adoption of specific practices. In this way, we were able to provide a comprehensive analysis of the laws and policies implemented during the early stages of the COVID-19 pandemic in relation to healthcare workers’ mobility across diverse regions.

## Results

Our study included an analysis of evidence of state practice in this regard in Argentina, Colombia, Kenya, South Africa, India, Japan, Spain, the United Kingdom, Canada, and the United States. The evidence includes government-published or government-broadcast content, such as diplomatic statements, international conduct and commitments, correspondence, verbal statements, and guidelines.

From March through April 2020, immediately following the WHO’s declaration of a public health emergency of international concern, these nations issued guidelines and verbal statements that freed healthcare workers from travel restrictions to expedite the delivery of medical supplies and service personnel.^1^

For instance, during a late March meeting with trade ministers from G20 countries, Indian Minister of Commerce and Industry Shri Piyush Goyal underscored a need to uphold multilateral commitments and create global procedures to ease the movement of medicines and healthcare professionals across national borders, stating: "We need to think of a suitable framework under which critical pharma products, medical devices, diagnostic equipment and kits and healthcare professionals can be deployed at short notice across territories under a pre-agreed protocol” [21].

Continental neighbors and G20 member states typically reacted in unison to safeguard thoroughfares for healthcare workers. For example, in Europe, Spanish President Pedro Sánchez explained at a March 22 press briefing that Spain along with EU member states agreed to a restriction on all non-essential travel from third countries for 30 days that “will not apply to ….healthcare workers and those who look after the elderly” [22]. The European Commission, on March 30, formally published guidance stating that a closure of the EU external border and restriction on travel “should not apply” to foreign nationals serving as healthcare professionals, health researchers, humanitarian aid workers, or transport personnel [23].

Likewise, in North America, when Canada on March 18 announced an exemption for U.S. healthcare providers from a suspension of travel across the Canadian-U.S. border, the U.S. reciprocated [24, 25].

Africa’s African Union (AU) Centres for Disease Control and Prevention on March 12 issued guidelines recommending exceptions for “personnel who perform essential functions, such as health and safety” from restrictions and precautions on AU meetings and travel to meetings in Africa [26].

In Asia, the foreign ministers of Japan, Korea, and China, during a teleconference, “agreed that it is extremely important” for G7 and G20 response actions to shore up “medical service delivery systems” in African countries and other developing countries, Japanese Foreign Minister Motegi Toshmitsu said at an April 17 press conference [27]. Later, at an Oct. 20 Japan-Indonesia Summit Meeting, Japan Prime Minister Suga Yoshihide, with Indonesia President Joko Widodo, affirmed that cross-border travel will resume for nurse and healthcare work candidates under the Japan-Indonesia Economic Partnership Agreement [28].

## Discussion

Healthcare workers’ freedom of movement during pandemics is essential under international law to ensure that individuals have access to adequate healthcare. The ability of healthcare workers to move freely across borders in times of pandemics allows for the sharing of knowledge and expertise, as well as the ability to provide healthcare in areas where it is needed most [29]. This can strengthen health systems by providing access to specialized care and increasing the capacity of healthcare providers to respond to outbreaks and other health crises.

Moreover, healthcare workers’ mobility is also a human rights issue. The right to health is recognized as a fundamental human right by the United Nations and is enshrined in the International Covenant on Economic, Social, and Cultural Rights. Access to healthcare is essential for the realization of this right, and healthcare workers’ mobility plays a critical role in ensuring that individuals have access to the care they need [30].

Additionally, healthcare workers’ mobility is an international cooperation issue. The WHO has recognized that the movement of healthcare workers is essential for the provision of healthcare services and that countries need to cooperate in order to ensure that healthcare workers are able to move freely across borders [8]. The WHO has also called for the removal of barriers to the movement of healthcare workers in order to facilitate the recruitment and deployment of healthcare professionals [31].

To strengthen healthcare systems during pandemics, it is important for governments to facilitate the movement of healthcare workers across borders. This can be done through the development of bilateral agreements between countries or through the establishment of regional mechanisms for the movement of healthcare workers during pandemics. International organizations, such as the WHO, can play a critical role in facilitating the movement of healthcare workers across borders. The WHO can provide technical assistance to countries to help them develop policies and programs to facilitate the movement of healthcare workers. Additionally, the WHO can work to build the capacity of healthcare workers through the provision of training and education programs.

To be sure, debates around healthcare workers mobility need to account for the “pervasive inequity in health workers distribution” and the threat it poses to the achievement of universal health coverage [7]. The global healthcare workforce faces a dynamic scenario where countries with low healthcare worker ratios often experience a significant drain of their staff to high-income countries, enticed by the promise of better salaries and living standards. This phenomenon, ultimately, amplifies existing disparities and inequities in healthcare access and quality. Nonetheless, while equity concerns persist in the broader context of healthcare workers mobility, the mobility observed during the COVID-19 pandemic presented a distinct situation. The mobility of healthcare workers during the pandemic was, based on our research, by and large, limited in scope. It was a short-term response to an immediate crisis, with the primary objective of providing critical care during the height of the pandemics. These movements were not driven by the pursuit of better salaries or living conditions but rather by a global commitment to combat the pandemic and save lives. Importantly, these healthcare workers were not granted long-term visas or residency in the countries they were supporting. Their presence was temporary, aligned with the acute phase of the pandemic. This approach, while reflecting the urgency of the situation, also ensured that healthcare workers mobility did not contribute to the traditional equity concerns associated with healthcare workers migration.

Overall, healthcare workers’ freedom of movement during pandemics is essential for ensuring that individuals have access to adequate healthcare, strengthening health systems, and protecting human rights. This conclusion is not only rooted in the imperative need to ensure that individuals can readily access quality healthcare services, but it also aligns seamlessly with the targets set forth in the Sustainable Development Goal 3. SDG 3 compels nations, international organizations, and healthcare professionals to work harmoniously to facilitate the unhindered movement of healthcare workers across borders. This collaboration is not merely an aspiration but an imperative for tackling communicable diseases and fostering well-being for people of all ages. The magnitude of this task extends beyond the confines of a single nation and necessitates the coordinated effort of all stakeholders, including governments, institutions, and the healthcare workforce itself. While Target 3.3 of the SDGs underpins the significance of international collaboration and collective action in promoting health on a global scale to end communicable diseases and promote well-being for all at all ages, Target 3.c focuses on the need to “substantially increase health financing and recruitment, development, training and retention of the health workforce in developing countries”.

Assessing the emergence of a norm of customary international law regarding healthcare workers’ freedom of movement is particularly appealing considering the various obstacles that hinder the adoption or effectiveness of international agreements, including states’ lack of political will and incentives to enter into such agreements. Customary international legal norms are an important source to draw from because once a norm develops, it becomes binding on states unless a state has persistently objected to the norm throughout the process of the norm’s creation [32].

Article 38 of the Statute of the International Court of Justice provides a statement of sources for international law, one of which is customary international law. Norms of customary international law materialize from uniform and consistent state practice taken under a state’s belief that it is compulsory as a matter of international law [32]. The subjective element of a customary international norm, referred to as *opinio juris,* is both necessary and elusive [33]. *Opinio juris* precludes a norm from merely developing out of usage as opposed to both usage and a belief, on behalf of a state, that such action is required as a matter of international law [33]. The majority view among scholars is that uniform and consistent state practice will not facilitate the creation of a binding norm if it is so motivated solely by political or economic interests as opposed to *opinio juris* [33].

It is important to address ways to identify customary international law and its elements—uniform and consistent state practice, as well as *opinio juris*—in order to detect state practice on the matter of healthcare workers’ freedom of movement. The International Law Commission (“ILC”) 2018 Draft Conclusions on the formation of customary international law (hereinafter “Draft Conclusions”) provide a discussion of each requirement of customary international law [34]. The Draft Conclusions dictate, under Conclusion Four, that the state practice “refers primarily to the practice of states that contributes to the formation or expression of rules of customary international law” [34]. Valid state practice, for purposes of determining the existence of a norm of customary international law, includes “conduct of the state, whether in the exercise of its executive, legislative, judicial or other functions;” “physical and verbal acts” as well as “inaction,” along with “diplomatic acts and correspondence, conduct in connection with resolutions adopted by an international organization or at an intergovernmental conference; conduct in connection with treaties; executive conduct;... legislative and administrative acts; and decisions of national courts” [34]. These forms of practice need be “sufficiently widespread and representative as well as consistent,” but not necessarily universal [34]. The Draft Conclusions expressly note that there is no duration requirement for state practice provided the practice meets the widespread and consistent requirements [34]. Part Four of the Draft Conclusions provides parameters for recognizing evidence of *opinio juris*, which the Draft Conclusions specify as meaning “[a]ccepted as law” [34]. *Opinio juris* may be ascertained through the following types of evidence, “public statements made on behalf of States; official publications; government legal opinions; diplomatic correspondence; decisions of national courts; treaty provisions; and conduct in connection with resolutions adopted by an international organization or at an intergovernmental conference” [34]. In accordance with the discussion above, the Draft Conclusions further instruct that while international organizations and intergovernmental conferences cannot create customary international law alone, resolutions adopted by these agencies “may provide evidence for determining the existence and content of a rule of customary international law, and contribute to its development,” and “[a] provision in a resolution adopted by an international organization or at an intergovernmental conference may *reflect* a rule of customary international law if it is established that the provision corresponds to a general practice that is accepted as law” [34].

Our findings showed that, within months, Argentina, Colombia, Kenya, South Africa, India, Japan, Spain, the United Kingdom, Canada, and the United States, ten nations representing most regions of the world, recognized, as law, the practice of excluding healthcare workers from prohibitions on movement during pandemics.

The findings indicate a strong consensus towards ensuring the free movement of healthcare workers during pandemics. To be sure, discerning *opinio juris* in connection with the instances of state practice described above is particularly difficult given the fact that the law in this surrounding *opinio juris* is unsettled. Thus, there is no dispositive answer as to whether the second component of a customary international norm compelling States to ensure healthcare workers’ freedom of movement during pandemics exists. In other words, it is not clear that the state practice on this matter was accompanied by a belief that such action was required as a matter of international law. Arguments can be made in favor of and against this conclusion; it would be imprudent to reach a definitive conclusion in such a short paper. Nonetheless, in general, it appears that evidence weighs in favor of the conclusion that a substantial amount of state practice was in fact taken under a sense of legal obligation or right.

## Conclusion

Healthcare workers’ freedom of movement was essential in the response to the COVID-19 pandemic. As the world prepares to respond to future pandemics or other health crises, it will be important for governments and international organizations to ensure healthcare workers’ mobility and include it in their preparedness and response plans.

As such, it becomes clear that the reform of the 2005 International Health Regulations and/or the adoption of a new pandemic treaty will bolster the strength of this emerging norm of customary international law and crystallize it. These legal instruments would propel a norm that is already in the process of formulation into existence [35]. Thus, crystallizing a norm that is otherwise emerging among states [35].

## Data Availability

With authors, upon request.

## Appendix

A March 19 Argentina decree created a quarantine exception for “health personnel.” Republic of Argentina. In: Federal Decree No. 297/2020. Official Gazette of the Republic of Argentina. 19 March 2020. https://www.argentinatexas.org/resources/Documents/CORONAVIRUS-Mandatory%20Quarantine%20Federal%20Decree%20No.%20297%202020%209ENG).pdf. Accessed 20 May 2023.

A March 20 Colombia ban on flights arriving and transiting via the country exempted individuals flying for a humanitarian emergency due to “force majeure or Act of God.” República de Colombia. In: Decreto Numero 439. 20 March 2020. https://dapre.presidencia.gov.co/normativa/normativa/DECRETO%20439%20DEL%2020%20DE%20MARZO%20DE%202020.pdf. Accessed 20 May 2023.

A related March 22 Colombia decree that prohibited free movement made an exception for international health organizations, health workers, and the construction of healthcare infrastructure to deal with the COVID health emergency. República de Colombia. In: Decreto Numero 457. 22 March 2020. https://dapre.presidencia.gov.co/normativa/normativa/DECRETO%20457%20DEL%2022%20DE%20MARZO%20DE%202020.pdf. Accessed 20 May 2023.

A March 25 Kenya curfew order that restricted individual and group movement did not apply to medical professionals and health workers. Kenya. In: The Public Order (State Curfew) Order. Kenya Subsidiary Legislation. 25 March 2020. https://covidlawlab.org/wp-content/uploads/2020/06/The-Public-Order-State-Curfew-Order-2020.pdf. Accessed 20 May 2023.

A March 25 South Africa rule prohibited the movement of persons and goods unless they involved medical, health, or laboratory services. Republic Of South Africa. In: Amendment of Regulations Issued in Terms of Sect. 27(s). Government Notices. No.R.398. 25 March 2020. https://www.gov.za/sites/default/files/gcis_document/202003/4314825-3cogta.pdf. Accessed 20 May 2023. The order also excluded individuals providing medical, health, or laboratory services from a ban on bus transport, taxi rides, e-hailing, and the use of private motor vehicles. Id.

A March 26 South Africa rule excluded workers providing such services from a prohibition on ferry transport and all long-distance and interprovincial means of travel. Republic of South Africa. In: Directions Issued In Terms Of Regulation 10(8) Of The Regulations Made In Under Sect. 27(2) Of The Disaster Management Act, 2002 (Act No. 57 Of 2002): Measures To Prevent And Combat The Spread of Covid -19 In The Public Transport Services. Government Notices. No. 412. 26 March 2020. https://www.gov.za/sites/default/files/gcis_document/202003/43157rg11065gon412.pdf. Accessed 20 May 2023.

A March 31 South Africa restriction on international and domestic flights permitted medical evacuation flights, along with aircraft operations involving humanitarian aid and relief. Republic Of South Africa. In: Amendment Of The Directions Issued In Terms Of Regulation 10(7) Of The Regulations Made In Under Section 27(2) Of The Disaster Management Act, 2002 (Act No. 57 Of 2002): Measures To Prevent And Combat The Spread Of Covid -19 In The Air Services. Government Notices. No. 438. 31 March 2020. Https://Www.Gov.Za/Sites/Default/Files/Gcis_Document/202003/43189gon438.Pdf. Accessed 20 May 2023.

March 24 India guidelines suspended air, train, rail, and all other transportation except for “[f]ire, law and order, and emergency services” transit. Government of India. In: Guidelines on the measures to be taken by Ministries/Departments of Government of India, State/Union Territory Governments and State/Union Territory Authorities for containment of COVID-19 Epidemic in the Country. Annexure to Ministry of Home Affairs Order No. 40–3/2020. 24 March 2020. https://ndma.gov.in/sites/default/files/PDF/covid/Guidelines.pdf. Accessed 21 May 2023.

In an April 7 video conference with Association of Southeast Asian Nations member states and the health ministers of China, Japan, and Korea, the nations pledged to coordinate cross-border public health responses, such as outbreak investigation, through existing bilateral and regional cooperation mechanisms. ASEAN Member States, People’s Republic of China, Japan, and the Republic of Korea. In: Adopted Joint Statement, Special Video Conference of ASEAN Plus Three Health Ministers in Enhancing Cooperation on Coronavirus Disease 2019 (COVID-19) Response. 7 April 2020, https://web.archive.org/web/20210126125747/https:/asean.org/storage/2020/04/Adopted-Joint-Statement_SVC.APTHMM_COVID-19.pdf. Accessed 21 May 2023.

A March 18 Canada order exempted foreigners “assisting in the COVID-19 coronavirus disease response” from a prohibition on foreigners flying into Canada from countries besides the U.S. Government of Canada. In: Orders in Council 2020–0157. 18 March 2020. https://orders-in-council.canada.ca/attachment.php?attach=38952&lang=en. Accessed 21 May 2023.

A March 24 Canada order created an exception for foreigners “providing medical care or transporting essential medical equipment, supplies, or means of treatment” from a 14-day isolation period upon entry into Canada. Government of Canada. In: Orders in Council 2020–0175. 24 March 2020. https://orders-in-council.canada.ca/attachment.php?attach=38989&lang=en. Accessed 21 May 2023.

A March 14 U.S. proclamation carved out an exception to a travel ban for foreigners entering from the U.K. or Ireland who are traveling for “a purpose related to containment or mitigation of the virus.” United States Government. Proclamation 9996—Suspension of Entry as Immigrants and Nonimmigrants of Certain Additional Persons Who Pose a Risk of Transmitting 2019 Novel Coronavirus. 14 March 2020. https://www.federalregister.gov/documents/2020/03/18/2020-05797/suspension-of-entry-as-immigrants-and-nonimmigrants-of-certain-additional-persons-who-pose-a-risk-of. Accessed 21 May 2023.

A March 24 U.S. prohibition barring the entry of foreigners from Canada exempted those traveling for “emergency response and public health purposes (e.g., government officials or emergency responders…)." United States Government. In: Notification of Temporary Travel Restrictions Applicable to Land Ports of Entry and Ferries Service Between the United States and Canada. 24 March 2020. https://www.federalregister.gov/documents/2020/03/24/2020-06217/notification-of-temporary-travel-restrictions-applicable-to-land-ports-of-entry-and-ferries-service. Accessed 21 May 2023.

An April 24 trilateral agreement with France, Ireland, and the U.K. committed the nations to protecting “[v]ital routes for supplies and people” to keep “the flow of goods and services running smoothly in and out of the UK, and around the country, throughout the pandemic.” United Kingdom. In: Vital routes for supplies and people kept open through coronavirus support package. 24 April 2020. https://www.gov.uk/government/news/vital-routes-for-supplies-and-people-kept-open-through-coronavirus-support-package. Accessed 21 May 21, 2023. A multimillion COVID support package facilitated essential freight services for critical routes; lifeline ferry services to the Isle of Wight and the Scilly Isles; and critical routes between Britain and the European mainland. Id. Also, the package extended to other European nations, including 26 routes between Britain, France, Belgium, Spain, the Netherlands, Denmark, Germany, Norway, and Sweden. Id.

A March 22 Spain rule that shut ports and airports to all foreigners allowed the entry of “[h]ealthcare professionals or those who look after the elderly travelling to exercise their profession.” Government of Spain. In: Government restricts access to travellers at Spain's external borders. Orders. 22 March 2020. https://www.lamoncloa.gob.es/lang/en/gobierno/news/Paginas/2020/20200322travellers.aspx. Accessed 21 May 2023.

## Abbreviations

AU: African Union
COVID-19: Coronavirus Disease 2019
EU: European Union
GATS: General Agreement on Trade in Services
GSHRH: Global Strategy on Human Resources for Health
G7: Group of Seven
G20: Group of Twenty
ILC: International Law Commission
SARS: Severe Acute Respiratory Syndrome
UHC: Universal Health Coverage
WHO: World Health Organization

## Footnotes

1 See Appendix.
